# Breast Sarcoidosis Management: A Case Report and Review of the Literature

**DOI:** 10.7759/cureus.97777

**Published:** 2025-11-25

**Authors:** Alshaimaa Alghamdi, Hanin M Alowaydhi, Fatamah F Kahtani, Zaher Mikwar

**Affiliations:** 1 General Surgery, King Abdulaziz Medical City, Ministry of National Guard Health Affairs, Jeddah, SAU; 2 General Surgery, King Saud Bin Abdulaziz University for Health Sciences, Jeddah, SAU; 3 General Surgery, King Abdullah International Medical Research Center, Riyadh, SAU; 4 College of Medicine, King Abdulaziz Medical City, Ministry of National Guard Health Affairs, Jeddah, SAU

**Keywords:** breast lesions, breast mass, breast sarcoidosis, granulomatous inflammation, lofgren’s syndrome, non-caseating granulomas, sarcoidosis diagnosis

## Abstract

Sarcoidosis is a systemic granulomatous inflammatory disease of unclear pathogenesis, predominantly affecting northern Europeans and occurring more commonly in females and non-smokers. Breast involvement in sarcoidosis is rare, often confused with malignant lesions, and typically diagnosed incidentally. This report presents a case of a breast lump initially suspected to be a recurrent abscess, later confirmed as sarcoidosis.

A 27-year-old breastfeeding mother presented with a large, painful right breast lump three months postpartum (P2G0). Initial imaging suggested an abscess or galactocele. Despite incision, drainage, and antibiotics, the mass recurred. Further surgical intervention and biopsy revealed non-caseating granulomas, consistent with sarcoidosis. Systemic involvement was ruled out through comprehensive imaging and laboratory tests, leading to a diagnosis of Löfgren’s syndrome. The patient was treated with systemic steroids and hydroxychloroquine, resulting in significant improvement and symptom resolution.

Breast sarcoidosis is often diagnosed incidentally owing to screening programmes. The literature review highlights the importance of tissue biopsy for definitive diagnosis, as clinical and radiological features are non-specific. Misdiagnosis with tuberculosis (TB) is common due to overlapping presentations. The relationship between sarcoidosis and cancer remains complex, with some studies suggesting an increased risk of malignancy in sarcoidosis patients.

Breast sarcoidosis, though rare, should be considered in the differential diagnosis of breast lesions. A multidisciplinary approach and thorough diagnostic workup are crucial. Further research is needed to better understand the potential malignancy risk associated with breast sarcoidosis.

## Introduction

Sarcoidosis is a systemic granulomatous inflammatory disease with an unclear pathogenesis [1]. Its incidence and prevalence are most highly reported in northern European countries, with an incidence of 60 per 100,000. It commonly affects individuals in their third and fourth decades of life. Regarding gender distribution, it is more common in females than in males, in non-smokers than smokers, and in certain racial groups such as African Americans [2,3].

Sarcoidosis is thought to result from an altered cellular immune response following exposure to environmental, infectious, or occupational hazards [4,5]. However, the pathophysiology is not fully understood. The relationship between infectious agents and the development of sarcoidosis has been investigated extensively to understand its probable etiology. Reports have described associations with Mycobacteria and other infectious agents such as *Borrelia burgdorferi* and Epstein-Barr virus (EBV). In addition, environmental exposures, including insecticides and molds, may contribute to the etiology of sarcoidosis [6].

Sarcoidosis is a diagnosis of exclusion, meaning it is diagnosed by ruling out other diseases that can cause similar clinical and histological features. This is particularly challenging due to its non-specific presentation. It manifests as a collection of symptoms when it infiltrates organs and causes fibrosis, such as the lungs, lymph nodes, skin, liver, spleen, eyes, and parotid glands. Lung involvement is the most common, occurring in approximately 90% of cases. Breast involvement, on the other hand, is extremely rare, accounting for about 1%, but when present, it may easily be confused with malignant or benign breast lesions [7].

The diagnosis is confirmed histopathologically by identifying non-caseating epithelioid granulomas, which may evolve into either complete resolution or hyaline fibrosis. Thus, the diagnostic criteria for sarcoidosis include a suggestive clinical and radiological context, histopathological evidence of non-caseating granulomas, and exclusion of other known causes of granulomatous disease such as tuberculosis (TB) [8,9]. As mentioned, breast involvement in sarcoidosis is uncommon and is usually discovered incidentally on screening mammography as a spiculated mass or via other imaging modalities. It may present as a painless mass, single or multiple, with variable size, possibly affecting one or both breasts, and with smooth or irregular edges. Such characteristics typically require further investigation to exclude malignancy [10,11]. In this paper, we present a case of a breast mass, initially suspected to represent a recurrent abscess, which was later diagnosed and appropriately managed as sarcoidosis.

## Case presentation

**Figure 1:**
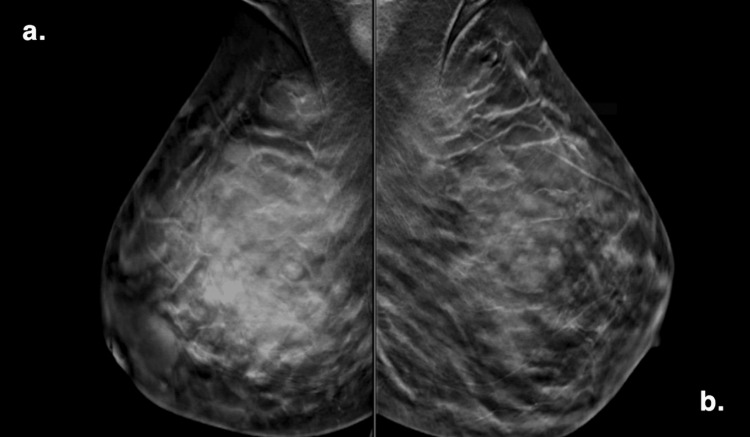
(a and b) Bilateral mammography showing a focal asymmetry in the superior aspect of the right breast, suspicious for an underlying mass.

A 27-year-old female (P2G0) with a body mass index of 29 and no significant medical history presented to the surgery clinic three months after the delivery of her baby with a large, painful, and erythematous right breast mass. She was vitally stable and afebrile. Examination of the right breast revealed marked redness, warmth, and a palpable 5 cm mass in the upper and retroareolar regions, accompanied by surrounding firmness. Additionally, there was an open wound at the 12 o’clock position, approximately 1 cm above the nipple-areola complex, measuring 1 cm in size and packed with a milky discharge. No palpable lymph nodes were detected in the right axilla. The left breast was unremarkable. Upon investigations, ultrasound (US) demonstrated a collection suggestive of an abscess or infected galactocele. A mammogram also suggested an infected galactocele. However, further discussion with the radiologist raised the possibility of inflammatory mastitis, ductal carcinoma in situ (DCIS), or sarcoidosis (Figure 1). Radiologists recommended continuing a prolonged course of antibiotics and considering breast MRI with vacuum-assisted biopsy if there was no improvement. As the patient did not improve, she underwent incision and drainage of the collection and was discharged on broad-spectrum antibiotics.

She later re-presented to the dressing clinic with a recurrent collection, and examination revealed two additional openings at the 7 o’clock position. She was planned for drainage under US guidance with interventional radiology, but repeat US identified multiloculated heterogeneous fluid collections. Consequently, surgical intervention for definitive drainage and biopsy was pursued. The patient also had non-healing gaping wounds from the previous procedure. Intraoperatively, multiple openings were noted at the 6, 3, and 7 o’clock positions of the right breast. A circumareolar incision was made, the collections were fully drained, and irrigation performed. Tissue samples were sent for biopsy and culture. (The initial collection ideally should have been sent for culture during the first incision and drainage.) The patient was discharged on antibiotics and instructed to continue daily dressing changes as an outpatient. Cultures were negative for bacteria, effectively ruling out an infectious etiology.

**Figure 2:**
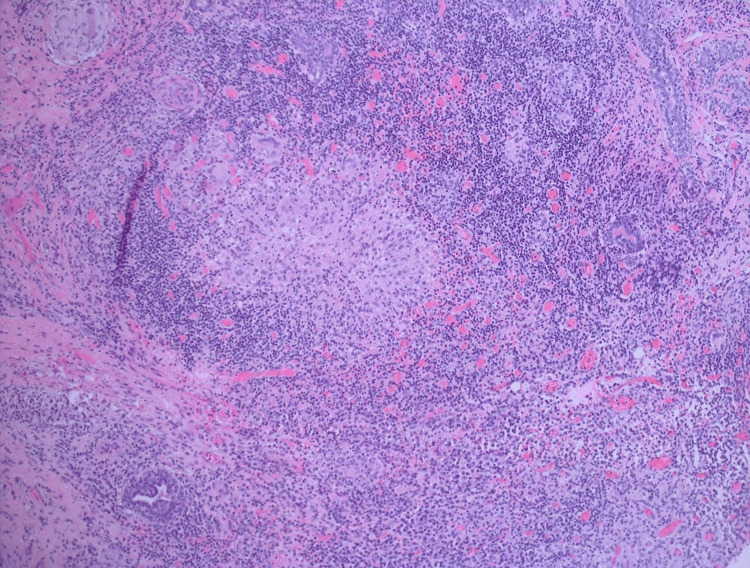
Histological features of a non-necrotizing granuloma surrounded by dense chronic inflammatory infiltrate (H&E, ×100).

**Figure 3:**
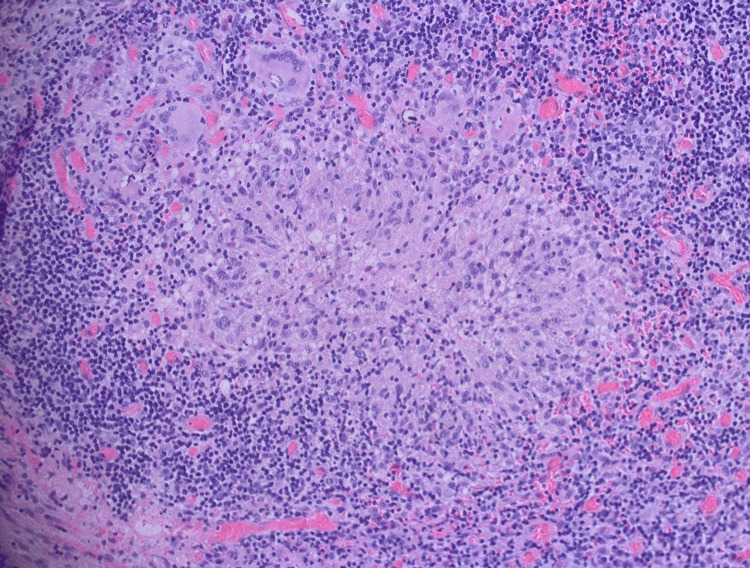
Histological features of a non-necrotizing granuloma surrounded by dense chronic inflammatory infiltrate and giant cells (H&E, ×200).

Final pathology revealed mammary tissue with lobulocentric granulomatous inflammation (Figures 2-3). Given the non-specific clinical features and histopathological findings, sarcoidosis was considered as a diagnosis of exclusion. A chest CT scan was performed to evaluate systemic involvement. The scan showed no intrathoracic disease and persistent right breast collections consistent with granulomatous mastitis. There was no hilar or axillary lymphadenopathy; subcentimeter mediastinal lymph nodes were noted, the largest measuring 0.4 cm in the right paratracheal region, not enlarged by CT criteria. The lung parenchyma showed a few small areas of subpleural nodularity in the left lung possibly representing focal atelectasis, with no definite pulmonary nodules. No pleural or pericardial effusion was seen, and major vascular structures and airways were patent. TB was excluded with an Interferon-Gamma Release Assay (IGRA) and chest X-ray, which showed no evidence of active or latent TB.

The patient was referred to rheumatology for further evaluation. She was diagnosed with sarcoidosis (Löfgren’s syndrome) based on hilar lymphadenopathy, skin manifestations, and ankle arthritis. She had elevated inflammatory markers, including ESR 99, angiotensin-converting enzyme (ACE) 53, and C-reactive protein (CRP) 171. She was also referred to ophthalmology and had no evidence of uveitis. She was started on systemic steroids and hydroxychloroquine. Her symptoms improved dramatically with no recurrence of the collection. She was last seen in the outpatient surgical clinic in January 2024, two years after her initial presentation, and all breast skin changes had completely resolved, with no further complaints. 

## Discussion

The first ever reported case was published in 1921. In early papers on breast sarcoidosis, almost 20% of cases presented with a breast mass as the main complaint, without any systemic manifestations. However, with the establishment of screening programs, incidental diagnosis has now become more common. In 2018, Chapelon-Abric C reviewed the literature and summarised twenty papers reporting breast involvement in sarcoidosis. Sarcoidosis was defined as a multisystem disease with two or more organs involved, with diagnosis confirmed by clinical symptoms, radiology, and tissue-proven pathology. Some papers highlighted the clinical relevance of high serum calcium or ACE levels in the diagnostic process. Among the twenty papers reviewed, fine-needle aspiration (FNA) was performed in only four studies, with positive findings in three out of four. Reported cytology showed lymphocytes, epithelioid-like cells, histiocytes, and reticulocytes without necrosis. However, tissue biopsy remains the standard of care. Several papers also described notable MRI features, such as microcalcifications and dilated ducts. One case mentioned in the review described a 42-year-old female with severe uncontrolled systemic sarcoidosis who underwent a fluorodeoxyglucose-positron emission tomography (FDG-PET) scan. The scan demonstrated a hypermetabolic breast lesion and the characteristic “butterfly” pattern of hypermetabolic mediastinal and hilar lymphadenopathy. In this instance, the breast hypermetabolism occurred simultaneously during a severe multisystemic flare of the disease, and regression of hypermetabolism following immunosuppressive therapy confirmed the diagnosis [2].

In a paper by Fiorucci F et al., the reported patient was 51 years old, and the need to exclude malignancy prompted an upfront excisional biopsy of the breast mass. The primary presentation included arthralgia and uveitis. During the workup, a suspicious breast mass was found incidentally. CT of the chest revealed bilateral hilar adenopathy, raising concern for either pulmonary involvement with primary breast cancer or systemic sarcoidosis. After reviewing the literature, the authors recommended excisional biopsy as a superior diagnostic procedure compared with FNA in such cases. This highlights the importance of obtaining a tissue biopsy to exclude alternative diagnoses in ambiguous presentations of breast masses [7].

In a Moroccan paper, the authors reported a 51-year-old female with a history of treated TB. She presented with non-specific symptoms of weight loss and fever and was admitted for workup with a query of TB. She was started on anti-TB medications but did not improve; therefore, a chest CT was performed, which revealed bilateral breast masses. Mammography showed right and left breast lumps classified as Breast Imaging Reporting and Data System (BI-RADS) V and BI-RADS IV, respectively. Biopsies were obtained, and the results demonstrated non-caseating granulomatous lesions. Their paper noted that a positive tuberculin skin test (TST) does not exclude sarcoidosis. As the patient had a history of treated TB, her TST was positive at 15 mm, and due to the high epidemiological prevalence of TB, it remained a leading differential diagnosis. This case highlights how nonspecific the presentation of sarcoidosis can be and how closely it may resemble other inflammatory systemic diseases such as TB. It underscores that laboratory testing alone is insufficient and that tissue diagnosis remains essential. Many cases of sarcoidosis are initially misdiagnosed as tuberculosis and vice versa. Both are granulomatous diseases that share similar clinical and radiological features, making distinction challenging. Corticosteroid therapy is the standard treatment for breast sarcoidosis and typically results in satisfactory clinical and radiological improvement. However, steroids can worsen symptoms in patients with latent TB if they are not receiving anti-TB therapy [11].

The association between sarcoidosis and cancer remains complex. However, patients with sarcoidosis appear to carry a higher risk of malignancy than the general population, particularly hematological and skin cancers. Because sarcoidosis can mimic breast cancer in its presentation, the question arises: does breast sarcoidosis increase the risk of developing breast cancer? The coexistence of both breast cancer and sarcoidosis has been reported in the literature. In a retrospective study conducted at the University of Southern California, 429 sarcoidosis patients were screened, and 20 of the 429 had a history of breast cancer. Among these, 12 patients were diagnosed with breast cancer before the diagnosis of sarcoidosis, 4 had long-standing sarcoidosis prior to the development of breast cancer, and the remainder were diagnosed with both conditions concurrently [12,13].

## Conclusions

Breast sarcoidosis is rare and can be difficult to distinguish from breast cancer or infected mastitis. Its existence must be recognised by clinicians, who should consider it in the differential diagnosis of breast lesions. The apparent rise in reported cases likely reflects increased breast screening rather than a true increase in disease prevalence. In this case report, and after reviewing similar cases, we recommend the following approach as a suggested method: First, involve a multidisciplinary team. Collaboration with specialties such as rheumatology, pulmonology, and infectious diseases allows broader discussion and consideration of a wider range of differentials. Second, diagnosis should be based on the patient’s clinical picture, imaging findings, and tissue biopsy, as tissue diagnosis is more sensitive than FNA. Lastly, there are no sensitive laboratory tests that reliably differentiate breast sarcoidosis from other conditions, and most inflammatory markers are nonspecific. In conclusion, excluding malignancy when suspected is essential. Further research into the risk of malignancy in breast sarcoidosis would be beneficial to clinical practice.
